# Exosomes secreted under hypoxia enhance stemness in Ewing’s sarcoma through miR-210 delivery

**DOI:** 10.18632/oncotarget.27702

**Published:** 2020-10-06

**Authors:** Matthew J. Kling, Nagendra K. Chaturvedi, Varun Kesherwani, Don W. Coulter, Timothy R. McGuire, J. Graham Sharp, Shantaram S. Joshi

**Affiliations:** ^1^Department of Genetics, Cell Biology and Anatomy, University of Nebraska Medical Center, Omaha, NE, USA; ^2^Department of Pediatrics, University of Nebraska Medical Center, Omaha, NE, USA; ^3^Department of Biomedical Sciences, Creighton University, Omaha, NE, USA; ^4^Department of Pharmacy Practice and Science, University of Nebraska Medical Center, Omaha, NE, USA; ^5^Child Health Research Institute, University of Nebraska Medical Center, Children’s Hospital of Omaha, NE, USA

**Keywords:** exosomes, hypoxia, Ewing’s sarcoma, stemness, miR-210

## Abstract

Intercellular communication between tumor cells within the hypoxic microenvironment promote aggressiveness and poor patient prognoses for reasons that remain unclear. Here we show that hypoxic Ewing’s sarcoma (EWS) cells release exosomes that promote sphere formation, a stem-like phenotype, in EWS cells by enhancing survival. Analysis of the hypoxic exosomal miRNA cargo identified a HIF-1α regulated miRNA, miR-210, as a potential mediator of sphere formation in cells exposed to hypoxic exosomes. Knockdown of HIF-1α in hypoxic EWS cells led to decreased exosomal miR-210 levels and reduced the capacity of hypoxic exosomes to form spheres. Inhibition of miR-210 in hypoxic spheres attenuated sphere formation and overexpression of miR-210 in normoxic spheres significantly enhanced the number of EWS spheres. Our results indicate that hypoxic exosomal miR-210 targets the proapoptotic protein CASP8AP2 in recipient cells. Moreover, the suppression of CASP8AP2 led to a reduction in apoptotic cells and increased sphere formation. Together, the findings in this study suggest that hypoxic exosomes promote stemness in EWS cells by delivering enriched miR-210 that is capable of down-regulating apoptotic pathways, resulting in the survival of cells with increased sphere formation. Future studies will further investigate the effects of EWS derived exosomal miRNAs on target genes and the role these interactions play in driving aggressiveness in hypoxic EWS tumors.

## INTRODUCTION

Ewing’s sarcoma (EWS) is an aggressive and highly malignant bone tumor that develops in children and adolescents [1]. In 85% of patients, EWS is driven by a chromosomal translocation, t [11, 22] (q24; q12), that results in the fusion protein, EWS-FLI1, which has been shown to regulate key pathways involved in EWS tumorigenesis [2, 3]. This pediatric tumor is treatable with a high degree of success if caught early, however, patients diagnosed with metastases or who relapse have a five-year survival rate of less than 25% and therefore, are considered to have the worst prognosis of all of the bone sarcomas [4]. These poor outcomes can be partly attributed to chemoresistant stem-like cancer cells [5]. Hypoxia is an essential feature of the tumor microenvironment (TME) in many solid tumors and it is suggested that enrichment of stem-like cells occurs within the hypoxic niche [6, 7], however, this mechanism remains poorly understood in EWS. Stabilization and activation of the hypoxia inducible factor HIF -1α in hypoxic tumor cells plays a major role in mediating the adaptive response to low oxygen levels [8]. HIF -1α has been demonstrated to regulate tumor formation and stem cell survival in hypoxic cancer cells by inhibiting apoptosis [9]. Emerging evidence indicates intercellular communication between tumor cells in hypoxic and normoxic regions contributes to functional differences associated with hypoxic tumors [10–12].

Recently, small extracellular vesicles called exosomes have been described as important mediators of cell-to-cell communication that transduce signals to neighboring cells and cells outside the local TME [11]. This function may provide a mechanism for how hypoxic cells communicate beyond their hypoxic niche and promote aggressive tumors. In the TME, exosomes range in size from 30–150 nm and carry bioactive lipids, protein, mRNAs, miRNAs and gDNA that can be horizontally transferred to cancer cells that drive tumor growth, progression, metastasis, drug resistance, angiogenesis and induction of pre-metastatic niches [13].

The role of exosomes in EWS has not been extensively explored. Initial reports focused on the mRNA profile in exosomes isolated from EWS cell lines and patient plasma where they identified a common transcript signature that could be used as biomarkers for detection of minimal residual disease [14]. Other reports demonstrated that shCD99 EWS-derived exosomes could transfer enriched miR-34a to recipient EWS cells and stimulate neural differentiation [15] while in another study, EWS-derived exosomes carrying *EZH2* mRNA could be delivered intact to mesenchymal stem cells [16].

Studies in other cancer models investigating the role of hypoxic exosomes have provided insight into how hypoxic tumors can secrete exosomes that propagate an aggressive phenotype in cells outside the hypoxic niche. Exosomes released from hypoxic prostate cancer cells enhanced sphere formation in normoxic cells [12], but the authors were unable to elucidate a mechanism describing how hypoxic exosomes promote stemness in normoxic cells. More recently, hypoxia was demonstrated to alter miRNA profiles in cancer-derived exosomes. Furthermore, exosomes have been shown to reprogram cancer cells, genetically and metabolically, through the delivery of their miRNA payload [11]. In breast cancer, hypoxic exosomes containing elevated levels of miR-210 increased migration and proliferation in recipient cancer cells [17]. In hypoxic tumors, miR-210 is directly regulated by HIF -1α at its hypoxia response element and is involved in driving metabolic changes, angiogenesis, cell proliferation, migration, invasion, apoptosis and stemness [18].

The role of hypoxia regulated exosomes and miRNA in EWS is currently unknown and highlights a major gap in scientific knowledge. Therefore, we sought to evaluate the role of hypoxic EWS-derived exosomes on the formation of stem-like cells in EWS. We hypothesized that the transfer of hypoxic derived exosomal miR-210 to normoxic cells silences the proapoptotic member caspase-8-associated protein 2 (CASP8AP2), resulting in increased sphere formation and survival.

## RESULTS

### Hypoxic^EXO^ enhance sphere formation in EWS cells

**Figure 1:**
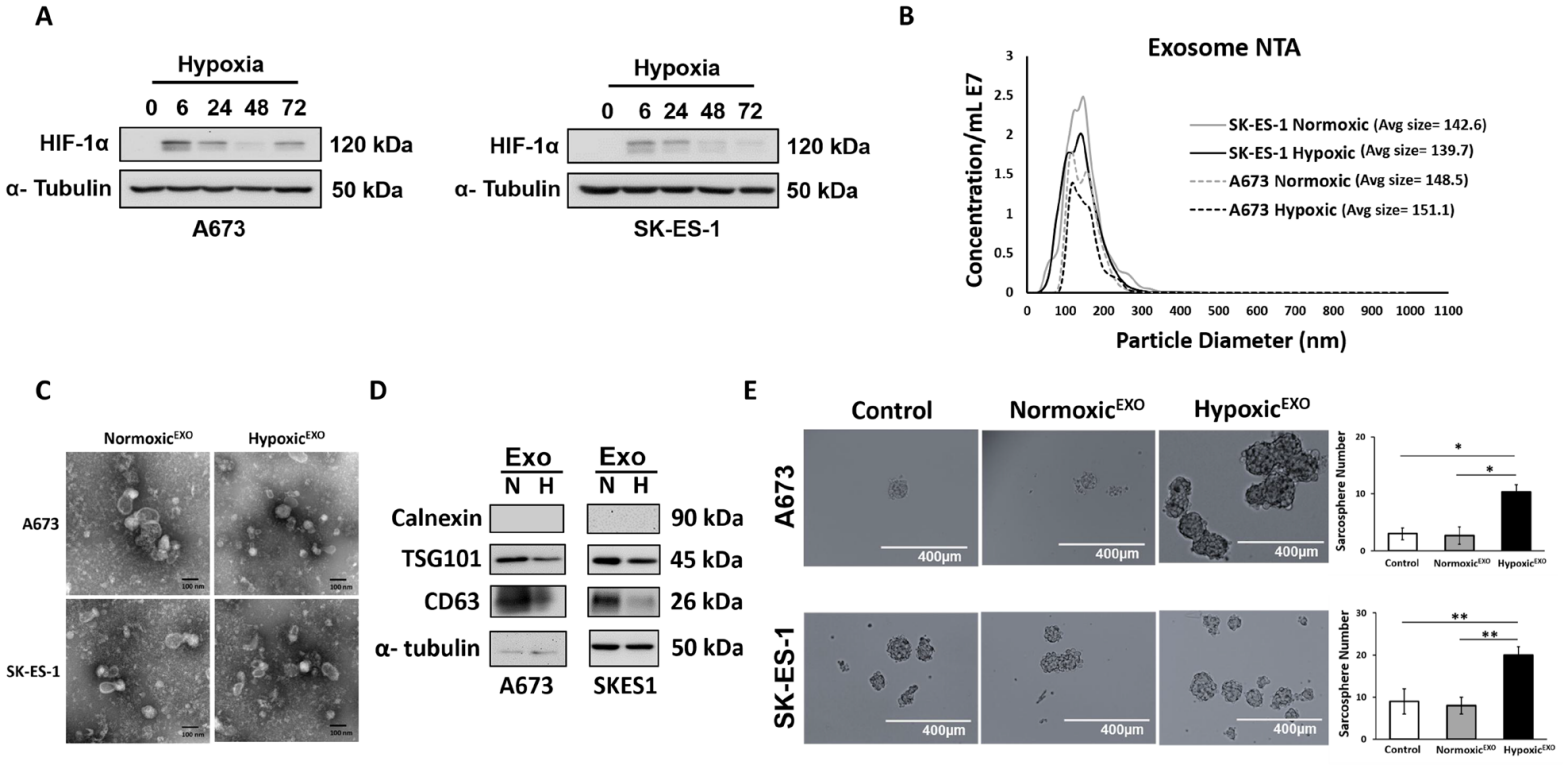
Hypoxic^EXO^ enhance sphere formation in EWS cells. (**A**) Western blot of HIF-1α in A673 and SK-ES- cells cultured under hypoxia (time points: 0, 6, 24, 48 and 72 hrs.). (**B**) NanoSight tracking analysis (NTA) of normoxic and hypoxic A673 and SK-ES-1 exosomes showing particle size and relative concentration. (**C**) Transmission electron micrograph (TEM) of normoxic and hypoxic A673 and SK-ES-1 exosomes. (**D**) Western blot of TSG101 and CD63 exosome markers and negative control calnexin. α-Tubulin was used as the internal loading control. (**E**) A673 and SK-ES-1 EWS cells were cultured in normoxic conditions with 20 μg/ml of their respective normoxic and hypoxic exosomes in a sphere assay. Quantification of spheres was performed (magnification, ×10) (Mean ± SEM, *n* = 3, ^*^ *P* ≤ 0.0008, ^**^ *P* ≤ 0.003).

Expression of HIF-1α was measured in A673 and SK-ES-1 EWS cells cultured under 1% O_2_ for an increasing duration. In both EWS cell lines, HIF -1α expression stabilized at 6 hrs (Figure 1A). To investigate the role of hypoxia on exosome secretion in EWS, A673 and SK-ES-1 cells were cultured in exosome-free media under normoxic (21%O_2_) and hypoxic (1%O_2_) conditions for 48 hours. EWS-derived exosomes were isolated by collecting the supernatant and performing multiple centrifugation and ultracentrifugation steps, followed by microfiltration. The exosomes were analyzed using Nanosight tracking analysis (NTA) to measure particle size and concentration. NTA revealed both A673 and SK-ES-1 exosomes with particle sizes within the expected size range of 30-150 nm (Figure 1B). In both cell lines, the total particle concentration of hypoxic EWS-derived exosomes (Hypoxic^EXO^) secreted was lower than normoxic EWS-derived exosomes (Normoxic^EXO^) following normalization to cell numbers. Proliferation rates in hypoxic EWS cells were decreased ~30% compared to normoxic cells which was consistent with the differences observed in particle concentration between normoxic and hypoxic cells. Total exosomal protein was also similar to total particle concentration and together, our findings suggest that EWS exosome secretion is dependent on proliferation rates which is consistent with the effects of hypoxia on cell proliferation.

EWS Normoxic^EXO^ and Hypoxic^EXO^ were visualized using transmission electron microscopy. TEM confirmed small vesicles ranging from 30–150 nm (Figure 1C). We confirmed the presence of EWS-derived exosomes by immunoblotting for protein markers involved in exosome biogenesis. Western blotting demonstrated decreased levels of CD63 and TSG101 in SK-ES-1 Hypoxic^EXO^ compared to Normoxic^EXO^. The endoplasmic reticulum protein calnexin served as a negative control and was not observed in either exosome lysates (Figure 1D). Total protein isolated from Normoxic^EXO^ and Hypoxic^EXO^ lysates was reflected in similar levels of α-tubulin. We concluded that our isolation method reliably captures vesicles consistent with exosome characteristics. We next investigated whether hypoxic exosomes promoted stemness in EWS cells outside of a hypoxic environment, we co-cultured EWS Normoxic^EXO^ and Hypoxic^EXO^ with normoxic cells in a sphere-forming assay. EWS cells cultured under normal oxygen tension levels demonstrated enhanced sphere formation after adding Hypoxic^EXO^ (Figure 1E). SK-ES-1 Hypoxic^EXO^ enhanced sphere formation 3.7-fold while A673 Hypoxic^EXO^ increased sphere formation by 2.2-fold compared to the effect of their respective Normoxic^EXO^ on normoxic cells. These results suggest that Hypoxic^EXO^ could enhance stemness in normoxic EWS cells.

### EWS Hypoxic^EXO^ carry specific hypoxia regulated miRNA

**Figure 2:**
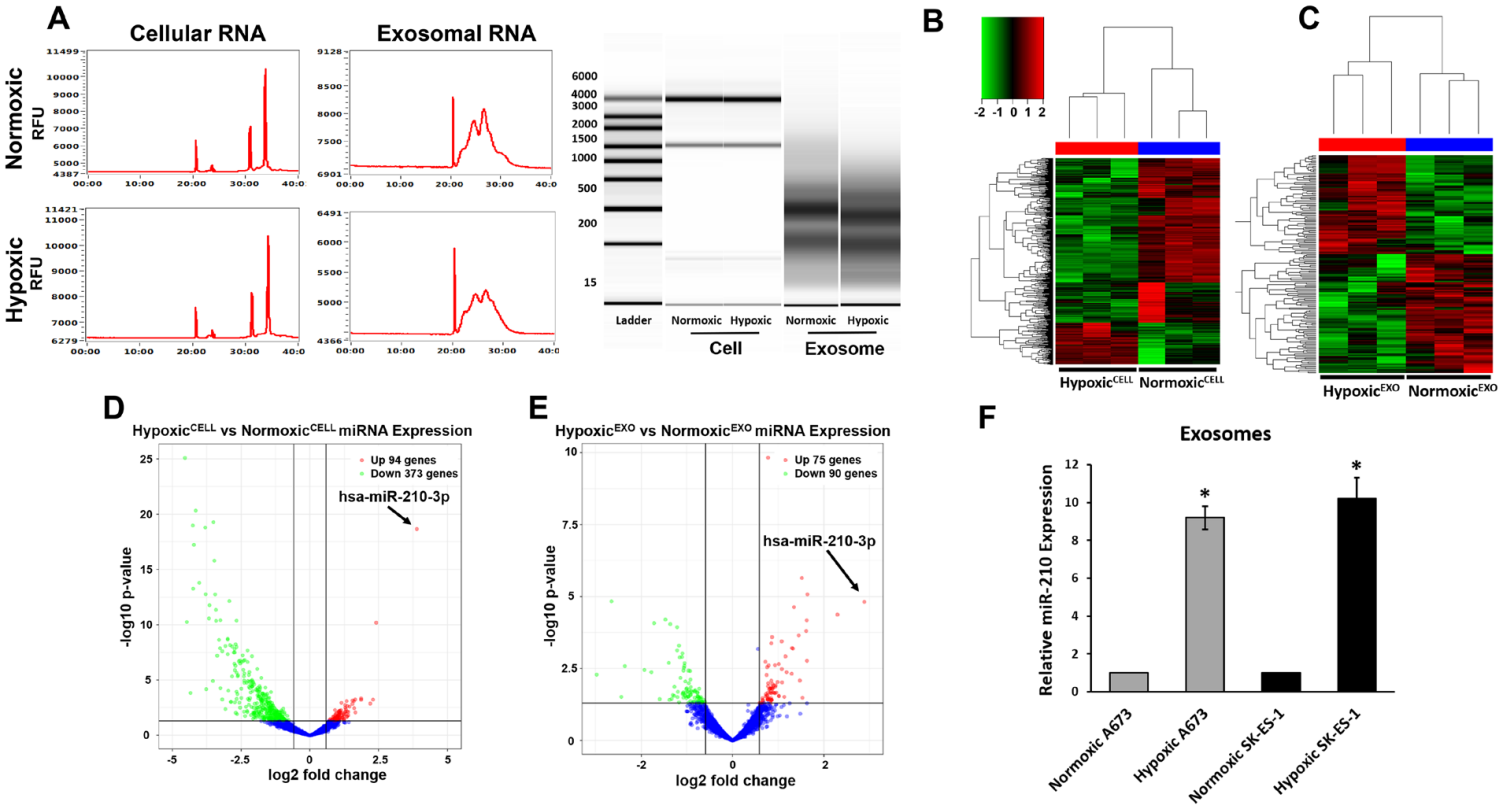
EWS Hypoxic^EXO^ carry specific hypoxia regulated miRNA. (**A**) Representative electropherogram profile of total RNA in normoxic and hypoxic SK-ES-1 cells and exosomes. Ribosomal subunits 28S and 18S were observed in cellular RNA profiles and were absent in exosomal RNA. Unsupervised cluster analysis of SK-ES-1 miRNAs in (**B**) normoxic and hypoxic cells and (**C**) Normoxic^EXO^ and Hypoxic^EXO^. Each column represents a biological replicate and every row denotes an individual miRNA. The Z-score color scale indicates high expression represented by red ranging to low expression denoted by green. Volcano plot of miRNA differentially expressed (≥ 1.5 fold threshold) in SK-ES-1 (**D**) normoxic compared to hypoxic cells and (**E**) Hypoxic^EXO^ compared to Normoxic^EXO^. Significance (-log10 *p-value*) was plotted versus fold change (log2 fold change) along the y and x axis, respectively. (**F**) Validation of hypoxia regulated miRNAs miR-210 A673 and SK-ES-1 Normoxic^EXO^ and Hypoxic^EXO^ using qRT-PCR (Mean ± SEM, *n* = 3, ^*^ *P* ≤ 0.05). miR-16 was used as a control to normalize miRNA expression.

Recently, hypoxia was demonstrated to alter miRNA profiles in cancer-derived exosomes. Furthermore, exosomes have been shown to genetically and metabolically reprogram cancer cells through the delivery of their miRNA payload [11]. We sought to identify miRNA in our Hypoxic^EXO^ that may play a role in promoting stemness. First, total RNA was isolated from normoxic and hypoxic SK-ES-1 cells and their derived exosomes. Capillary electrophoresis revealed a broad composition of RNA with enrichment of small RNAs in the exosomes compared to their respective cells (Figure 2A). Cellular RNA profiles demonstrated typical ribosomal subunit peaks (28S and 18S), but were not seen in the exosomal lysates [19]. The presence of these ribosomal RNAs have been reported in both microvesicles and apoptotic bodies which further supports the high quality of the exosome isolation.

RNAseq was performed using the Illumina HiSeq3000 to identify exosomal miRNAs. Unsupervised hierarchical clustering identified miRNA clusters that were significantly different between normoxic and hypoxic cells, and Normoxic^EXO^ and Hypoxic^EXO^ (Figure 2B, 2C). We observed 467 miRNAs to be differentially expressed between normoxic and hypoxic cells with a threshold for comparison of 1.5 fold with a significance of *P* < 0.05) (Figure 2D). Hypoxic cells displayed 94 increased and 373 decreased miRNAs compared to normoxic cells. In Hypoxic^EXO^, we observed 75 enriched and 90 depleted miRNAs compared to Normoxic^EXO^ (Figure 2E). Using real-time PCR, we validated the top regulated miRNA observed in both the cellular and exosomal miRNA-seq results and found miR-210 expression to be consistently elevated in Hypoxic^EXO^ compared to Normoxic^EXO^ for both EWS cell lines (Figure 2F). These data suggest that hypoxic exosomal miR-210 may influence sphere formation in target cells and could potentially serve as a reliable prognostic marker for hypoxic EWS tumors.

### HIF-1α upregulates exosomal miR-210 and mediates Hypoxic^EXO^-induced sphere formation

**Figure 3:**
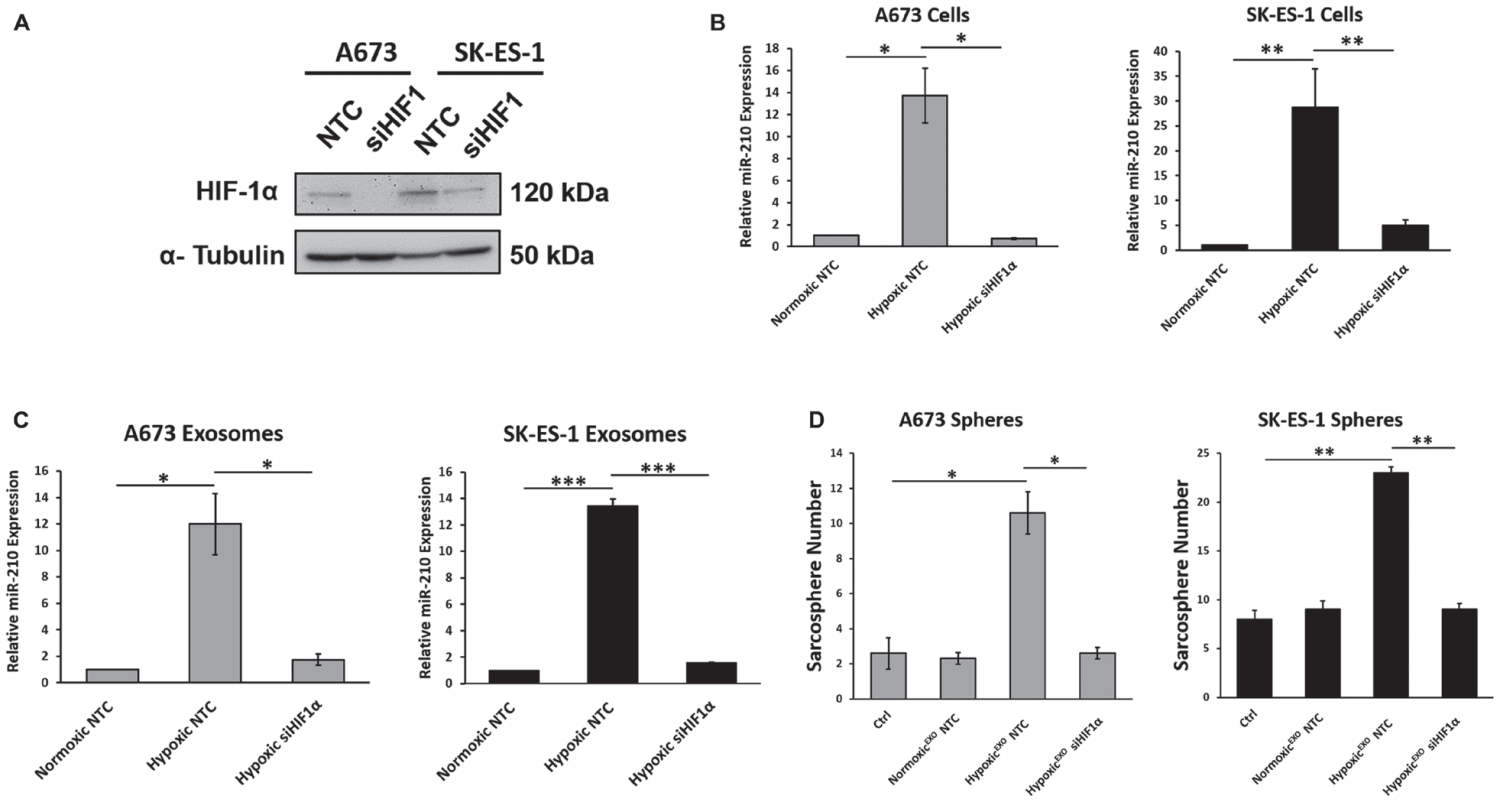
HIF-1α upregulates exosomal miR-210 and mediates Hypoxic^EXO^-induced sphere formation. (**A**) Western blot of HIF-1α in hypoxic A673 and SK-ES- cells transfected with Non-Targeting control (NTC) and HIF-1α-siRNA. α-Tubulin was used as the internal loading control. (**B**) Analysis of miR-210 expression levels in A673 and SK-ES-1 cells transfected with NTC and HIF-1α-siRNA, and (**C**) their respective exosomes using qRT-PCR (Mean ± SEM, *n* = 3, ^*^ *P* ≤ 0.02, ^**^ *P* ≤ 0.05, ^***^ *P* ≤ 0.0002). RNU6B and miR-16 was used as a control to normalize miRNA expression in cells and exosomes, respectively. (**D**) Assessment of sphere formation in A673 and SK-ES-1 EWS cells cultured in normoxic conditions with 20 μg/ml normoxic and hypoxic exosomes derived from their respective cells transfected with NTC and HIF-1α-siRNA. Quantification of spheres was performed (Mean ± SEM, *n* = 3, ^*^ *P* ≤ 0.0004, ^**^ *P* ≤ 0.0001).

Since HIF-1α has been shown to regulate the expression of miR-210 in hypoxic cells, we evaluated whether miR-210 levels in hypoxic EWS cells and their Hypoxic^EXO^ were HIF-1α dependent by knocking down HIF-1α using siRNAs (siHIF-1α) (Figure 3A). In our HIF-1α knockdown cells, we observed a significant decrease in HIF-1α under hypoxic conditions. Knockdown of HIF-1α significantly decreased miR-210 in both our hypoxic cells (Figure 3B) and in their Hypoxic^EXO^ (Figure 3C). In addition, miR-210 levels in the HIF-1α knockdown cells and exosomes were similar to their normoxic controls. These results indicate that miR-210 expression in hypoxic EWS cells and Hypoxic^EXO^ is regulated by HIF-1α. Next, we assessed the effect of Hypoxic^EXO^ derived from siHIF-1α cells on sphere formation (Figure 3D). Here, Hypoxic^EXO^ derived from siHIF-1α cells failed to increase sphere formation compared to control Hypoxic^EXO^. Together, these findings suggest that the stabilization of HIF-1α in hypoxic cells plays a significant role in the Hypoxic^EXO^ mediated effects on sphere formation.

### miR-210 in Hypoxic^EXO^ increases EWS sphere formation

**Figure 4:**
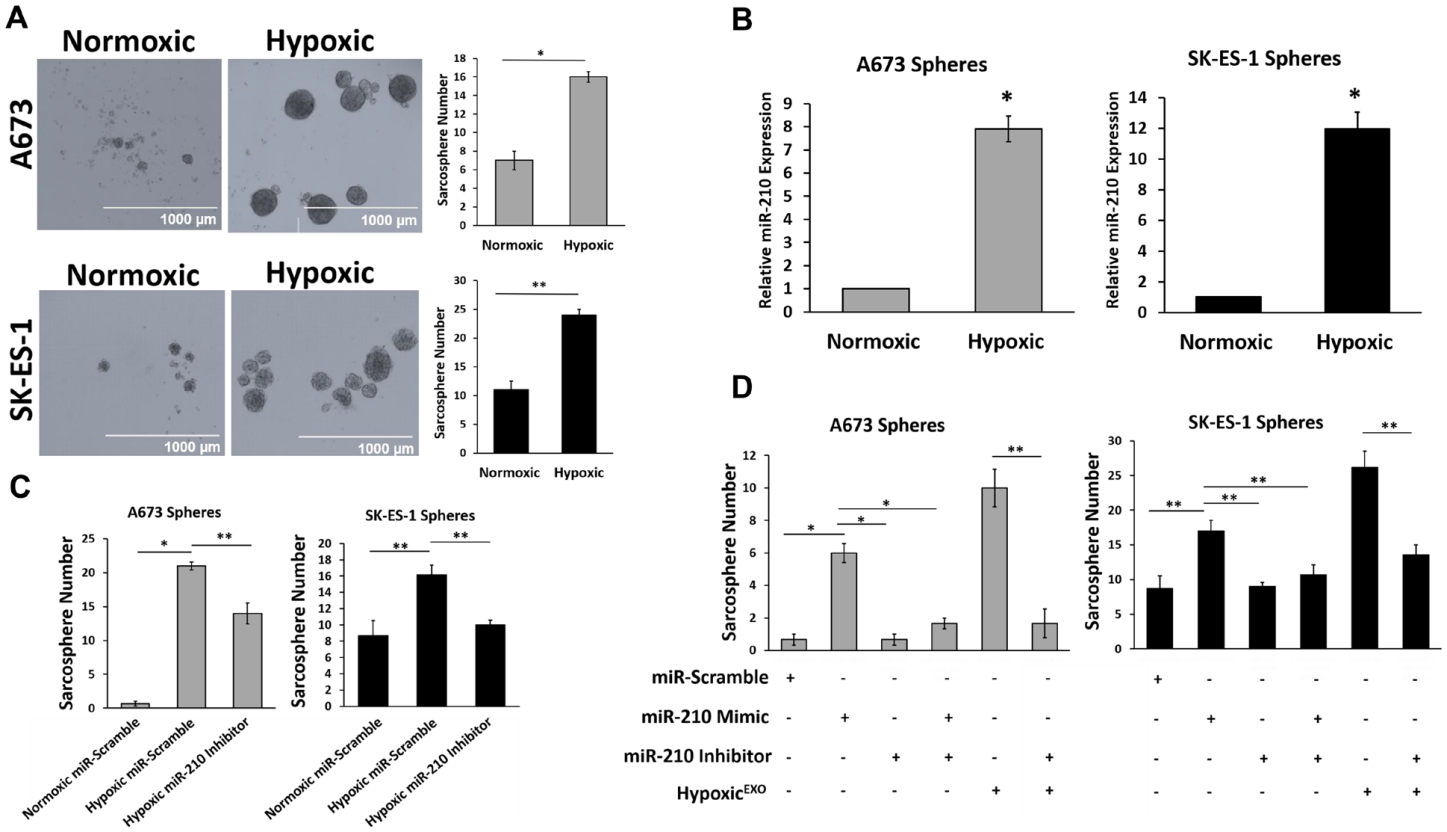
miR-210 in Hypoxic^EXO^ increase EWS sphere formation. (**A**) Sphere assay of A673 and SK-ES-1 cells grown under normoxic and hypoxic conditions. Quantification of spheres was performed (magnification, ×4) (mean + SEM, *n* = 3, ^*^ *P* ≤ 0.02, ^**^ *P <* 0.008). (**B**) Analysis of miR-210 expression levels in normoxic and hypoxic A673 and SK-ES-1 spheres using qRT-PCR (Mean + SEM, *n* = 3, ^*^ *P* ≤ 0.5). RNU6B was used as a control to normalize miRNA expression. (**C**) Sphere assay quantifying A673 and SK-ES-1 cells transfected with either miR-scramble or miR-210 inhibitors under normoxic and hypoxic conditions. (Mean + SEM, *n* = 3, ^*^ *P* ≤ 0.0001, ^**^ *P* ≤ 0.005). (**D**) Sphere assay quantifying A673 and SK-ES-1 cells transfected with miR-scramble, miR-210 mimics, miR-210 inhibitors or 20 μg/ml hypoxic exosomes. (Mean + SEM, *n* = 3, ^*^ *P* ≤ 0.002, ^**^ *P* ≤ 0.0001).

To determine if hypoxic exosomal delivery of miR-210 increases sphere formation, we first needed to measure the effects of hypoxia on sphere formation in EWS cells. EWS cells cultured under hypoxic conditions formed significantly more and larger spheres than cells cultured under normoxia (Figure 4A). Hypoxic spheres expressed significantly elevated levels of miR-210 compared to normoxic spheres (Figure 4B). To study if miR-210 is required for sphere formation in EWS cells, we knocked down miR-210 using inhibitors and overexpressed miR-210 using mimics (Figure 4C and 4D). Inhibition of miR-210 in hypoxic spheres resulted in a significant decrease in sphere formation in both EWS cell lines, however, while the number of spheres formed in hypoxic SK-ES-1 knockdown cells was similar to normoxic spheres, hypoxic A673 knockdown cells had an elevated sphere formation compared to their normoxic spheres. The overexpression of miR-210 in normoxic EWS cells promoted a 9-fold and 2-fold increase in spheres formed in A673 and SK-ES-1 cells, respectively. Normoxic spheres transfected with miR-210 inhibitors or combined mimics with inhibitors demonstrated no significant change in sphere formation in either EWS cell line.

To evaluate the role of miR-210 in Hypoxic^EXO^ mediated sphere formation, we treated normoxic EWS cells with Hypoxic^EXO^ alone and in the presence of cells transfected with miR-210 inhibitors (Figure 4D). The effects of Hypoxic^EXO^ on sphere formation were significantly impaired in both A673 and SK-ES-1 cells treated with the miR-210 inhibitor. Together, these results indicated that miR-210 in hypoxic spheres and Hypoxic^EXO^ plays a causal role in increasing stemness in EWS cells.

### miR-210 silences the proapoptosis member CASP8AP2

**Figure 5:**
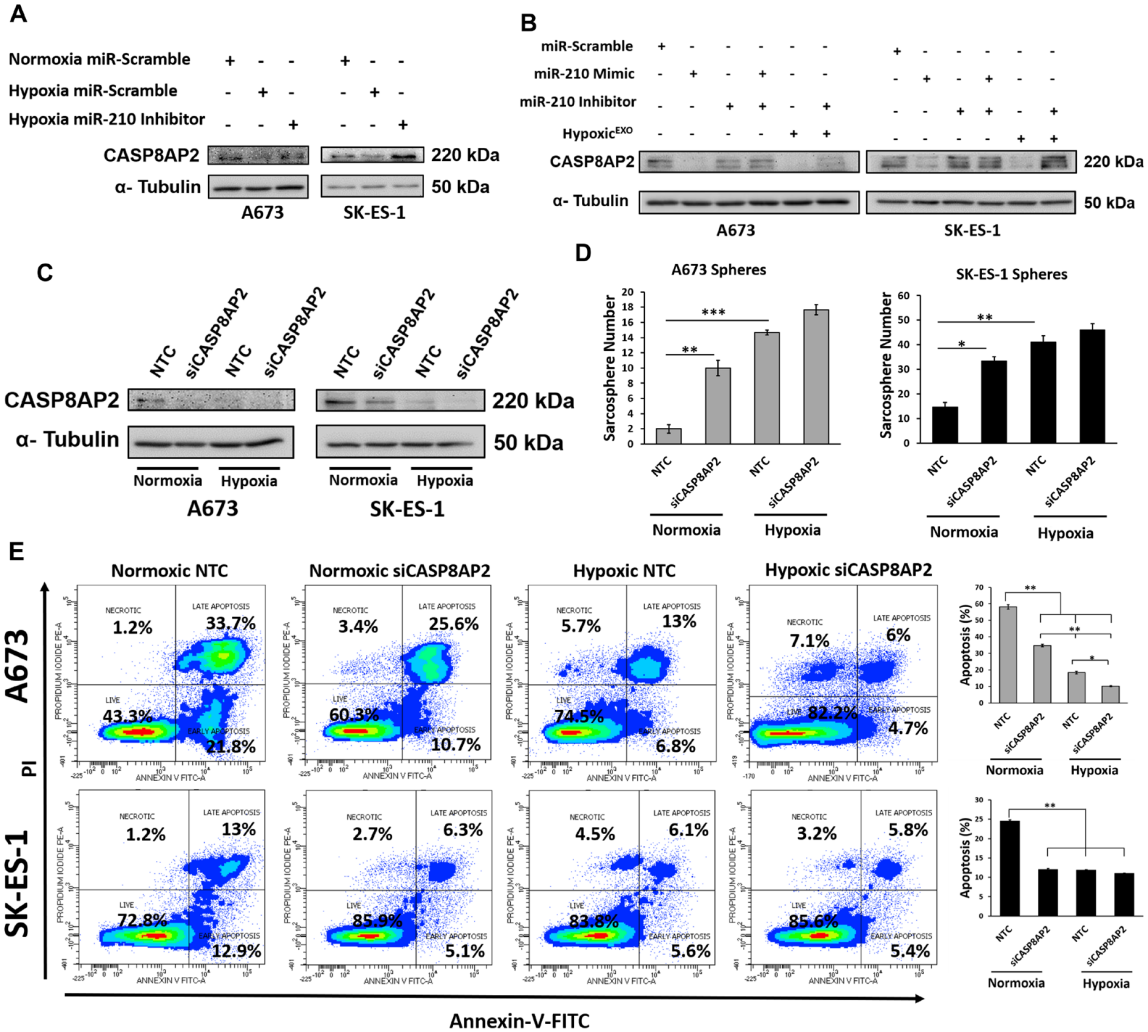
miR-210 silences the proapoptosis member CASP8AP2. Western blot of CASP8AP2 in (**A**) A673 and SK-ES-1 Spheres transfected with either miR-scramble or miR-210 inhibitors under normoxic and hypoxic conditions. (**B**) A673 and SK-ES-1 spheres transfected with miR-scramble, miR-210 mimics, miR-210 inhibitors or 20 μg/ml hypoxic exosomes. (**C**) A673 and SK-ES-1 spheres transfected with Non-Targeting control (NTC) or CASP8AP2-siRNA. α-Tubulin was used as the internal loading control. (**D**) Sphere assay quantifying A673 and SK-ES-1 cells transfected with either NTC or CASP8AP2-siRNA under normoxic and hypoxic conditions. (Mean + SEM, *n* = 3, ^*^ *P* ≤ 0.002, ^**^ *P* ≤ 0.0002, ^***^ *P* ≤ 0.0001). (**E**) Assessment of apoptosis by Annexin V-FITC/PI staining. (Left) Representative plots and (Right) Quantification of Annexin V-FITC stainined A673 and SK-ES-1 spheres transfected with NTC or CASP8AP2-siRNA under normoxic and hypoxic conditions. (Mean + SEM, *n* = 3, ^*^ *P* ≤ 0.0008, ^**^ *P* ≤ 0.0001).

To identify a functional mechanism underlying the effects of miR-210 on sphere formation, we analyzed a known miR-210 target gene, the proapoptotic member CASP8AP2. Previous studies utilized miRNA target prediction tools (miRANDA, Sanger MirBase and Targetscan) to identify a miR-210 conserved seed sequence in the CASP8AP2 3′-UTR region [20, 21]. The miR-210 putative target sites in CASP8AP2 were validated using luciferase activity assays and functional studies revealed a prosurvival effect resulting from miR-210 directly silencing CASP8AP2 [20–23]. In our hypoxic EWS spheres, expression of CASP8AP2 was significantly decreased compared to normoxic spheres and inhibition of miR-210 in hypoxic spheres restored CASP8AP2 expression when compared to normoxic spheres (Figure 5A). Overexpression of miR-210 decreased CASP8AP2 in normoxic spheres when compared to normoxic controls (Figure 5B). As expected, CASP8AP2 expression was rescued in normoxic spheres cotransfected with miR-210 mimics and inhibitors. Importantly, normoxic spheres treated with Hypoxic^EXO^ decreased CASP8AP2 levels, while miR-210 reversed the exosome mediated downregulation of CASP8AP2 (Figure 5B). To demonstrate a direct effect of CASP8AP2 expression on sphere formation, we knocked down CASP8AP2 using siRNAs (Figure 5C). CASP8AP2 knockdown led to significant increases in sphere formation in normoxic cells but had little effect on hypoxic spheres when compared to hypoxic controls (Figure 5D). To further investigate the functional role of CASP8AP2 in the survival of EWS spheres, we assessed apoptosis using Annexin V/PI staining and flow cytometry (Figure 5E). Inhibition of CASP8AP2 in normoxic spheres significantly reduced apoptosis compared to normoxic controls in both cell lines. Interestingly, SK-ES-1 hypoxic control and CASP8AP2 knockdown spheres had similar reductions in apoptosis as the normoxic CASP8AP2 knockdowns, whereas, A673 hypoxic controls demonstrated a further decrease in apoptosis compared to normoxic CASP8AP2 knockdowns. Moreover, CASP8AP2 knockdown further decreased apoptosis in hypoxic spheres compared to the hypoxic control group. Our findings suggest that Hypoxic^EXO^ mediated delivery of miR-210 to normoxic EWS spheres mediates the silencing of CASP8AP2 and promotes survival and stemness in EWS cells.

## DISCUSSION

The hypoxic TME is considered a crucial driver in tumor development and therapy resistance. Aggressive tumors often consist of hypoxic regions capable of creating an environment that supports stem-like cancer cells [6, 7]. In EWS, hypoxia and enrichment of stem-like cells is associated with poor patient outcomes [5, 24]. Mounting evidence suggests that the hypoxic niche may be able to exert its effects beyond the local microenvironment by signaling to nearby normoxic tumor cells [11]. Exosomes have been described as critical mediators of cell-cell communication within the TME and may provide an explanation for how hypoxia contributes to tumors with an aggressive phenotype. Our findings indicate that exosomes secreted under hypoxic conditions significantly increase sphere formation in normoxic EWS cells. Moreover, we show that HIF-1α regulates the Hypoxic^EXO^ mediated effect on sphere formation by enriching miR-210 within hypoxic derived exosomes. We demonstrate that the delivery of miR-210 to recipient cells enhances sphere formation by silencing a potential target, the proapoptotic member CASP8AP2, that is critical to the survival of stem-like cells.

Regions within tumors experiencing prolonged hypoxia result in necrosis and are linked to poor patient survival. A clinical study in EWS reported primary tumors with necrotic regions correlated with the worst overall survival in patients who had increased chances of metastases [24]. Recently, a growing body of evidence has shown that exosomes function as a bridge between neighboring cancer cells by delivering an RNA rich cargo [11, 25, 26]. In hypoxic tumors, exosomes have been suggested to contribute to the aggressive phenotype by mediating the cross-talk between tumor cells in hypoxic regions and target cells in normoxic regions [11, 12]. However, the association between hypoxia and exosomes in EWS has yet to be explored. Initial studies demonstrated that Hypoxic^EXO^ were being secreted at an elevated rate compared to normoxic cells and was suggested to underly the effects observed in cells exposed to Hypoxic^EXO^ [27]. Our findings and others indicate that exocytosis of exosomes is dependent on cellular proliferation, explaining our results demonstrating a decrease in Hypoxic^EXO^ secretion [12]. Consistent with this report, our observed Hypoxic^EXO^ mediated effects on stemness are most likely due to the exosomal cargo, not hypersecretion. These discrepancies in exosomal secretion could be attributed to the use of exosome isolation methods that poorly discriminate exosomes from microvesicles or apoptotic bodies, however, no isolation approach can totally eliminate these extracellular vesicles. Furthermore, RNA analysis in our exosomes lacked ribosomal peaks which are commonly observed in microvesicles and apoptotic bodies [19].

In cancer patients, exosomes are reportedly elevated within the systemic vasculature compared to normal healthy control patients and carry biomarkers with the potential to detect and monitor the development of tumors [13]. Characterization of mRNA in EWS cells and their derived exosomes was previously performed in order to identify an RNA profile that could be used to detect minimal residual disease following therapy [14]. In our approach, we assessed the miRNA expression profiles in hypoxic and normoxic EWS cells and their derived exosomes. Importantly, the differential expression of our exosomal miRNA cargo revealed selective sorting of small RNAs in Hypoxic^EXO^ that in part, reflected the miRNA profile in our hypoxic cells. Detection of hypoxia regulated miRNAs in circulating exosomes have played a major role as prognostic and predictive markers that correlate with metastasis and chemoresistance [27, 28]. We validated the top miRNA, miR-210, that was differentially expressed in our hypoxic cells and Hypoxic^EXO^. These observations are consistent with findings in exosomes derived from hypoxic breast cancer cells and patient samples [27, 29]. Moreover, breast cancer patients with elevated exosomal miR-210 were associated with tumor metastasis [30]. In EWS patients, EWS-FLI1 is the sole diagnostic marker that distinguishes EWS from other sarcomas and bone cancers [3]. In our study, we are the first to show a miRNA profile in EWS exosomes that could serve as biomarkers and assist in the prediction of outcomes in EWS patients with aggressive hypoxic tumors. Future work validating and matching the molecular profiles between exosomes and their parental EWS tumors is needed in order to identify EWS patients with hypoxic tumors and to devise new approaches that can overcome therapy resistant tumors.

In addition to exosomal cargo serving as biomarkers, increasing efforts are being made in elucidating the mechanism’s underlying exosomal miRNAs silencing of target mRNAs in recipient cells, prompting our investigation into the miRNA profile in EWS exosomes. Recent studies in breast cancer revealed exosomal miRNA promoted tumorigenesis in a Dicer-dependent manner and moreover, Dicer was demonstrated to facilitate miRNA processing from pre-miRNA to mature miRNA in exosomes resulting in stable miRNA capable of exerting long lasting pro-tumorigenic effects in recipient cells [31]. Several studies have identified mechanisms describing the effects of Hypoxic^EXO^ miRNA cargo in angiogenesis, metastasis, proliferation, migration and invasion, but it is currently unknown how Hypoxic^EXO^ delivery of miRNA influences stemness in recipient cells. A previous study indicated exosomes derived from hypoxic prostate cancer cells can enhance sphere formation in normoxic cells [12], however, the underlying molecular mechanism remains unclear. Since HIF-1α is a primary regulator of the hypoxic response, an early study reported enrichment of miR-21 in exosomes derived from hypoxic oral squamous cell carcinoma cells was directly regulated by stable HIF-1α [11]. In our study, we demonstrate that the effects of Hypoxic^EXO^ on sphere formation in normoxic EWS cells is regulated by HIF-1α remodeling of the Hypoxic^EXO^ miRNA cargo and directly enriching for miR-210. This is supported by our observations showing Hypoxic^EXO^ derived from HIF-1α knockdown cells failed to increase sphere formation compared to normoxic control spheres and spheres co-cultured with Normoxic^EXO^. Given that miR-210 was highly expressed in both our hypoxic cells and exosomes, we are the first to show that increased miR-210 levels in Hypoxic^EXO^ are dependent on HIF-1α stabilization. Together, this evidence compelled us to investigate the role of cellular and exosomal miR-210 in promoting stemness in EWS cells.

It is well established that a myriad of miRNAs are capable of targeting overlapping mRNA in cells, especially in recipient cells that have internalized cancer derived exosomes [13, 31, 32]. Our observed effects of Hypoxic^EXO^ miRNA content on normoxic spheres might not be the result of a single miRNA, however, our findings indicate that miR-210, is at least one miRNA capable of mediating sphere formation in hypoxic spheres and in spheres co-cultured with Hypoxic^EXO^. Previously, knockdown of miR-210 in hypoxic glioma stem cells was demonstrated to decrease sphere formation and the overall stem cell phenotype [33]. In osteosarcoma stem cells, miR-210 overexpression and knockdown studies revealed that miR-210 can promote stemness and even reprogram differentiated osteosarcoma cells into Stro-1^+^/CD117^+^ stem cells [34]. Our study also demonstrated that inhibition of miR-210 could reduce sphere formation in hypoxic spheres. Moreover, when we overexpressed miR-210 in normoxic EWS spheres, it resulted in increased sphere formation consistent with previous studies demonstrating a miR-210 mediated effect on cellular reprogramming [34]. Importantly, we are the first to show in EWS that hypoxia increases sphere formation and in part, sphere formation is regulated by miR-210 expression. In breast cancer, hypoxic derived exosomal miR-210 promoted angiogenesis and proliferation in xenograft tumors [17]. Delivery of Hypoxic^EXO^ to normoxic cells in our study indicated that exosomal miR-210 can promote sphere formation. Furthermore, we show that miR-210 can enhance stemness by downregulating CASP8AP2.

This is the first study to describe a mechanism where the miRNA cargo in Hypoxic^EXO^ silence a gene involved in regulating the survival of stem-like cells. In hypoxic bone marrow derived mesenchymal stem cells (BM-MSC), miR-210 targeted and silenced CASP8AP2, resulting in an antiapoptotic affect that bolstered the survival of the BM-MSCs [20]. CASP8AP2 is a proapoptotic member that participates in fas-induced and tumor necrosis factor-α- mediated apoptosis signaling [35]. Since EWS cells are thought to derive from transformed BM-MSCs, we chose to investigate if EWS cells can adopt this prosurvival pathway to preserve its stem-like population. Consistent with previous findings, knockdown of miR-210 in our hypoxic spheres resulted in increased CASPA8AP2 expression, while overexpression of miR-210 in normoxic spheres inhibited CASP8AP2 levels [20–23, 36]. Hypoxic^EXO^ delivering miR-210 demonstrated a similar decrease in CASP8AP2 expression in normoxic spheres. miR-210 exerts pleiotropic antiapoptotic effects on hypoxic cells [20, 33, 37, 38], however, this is the only study in EWS demonstrating a mechanism where miR-210 promotes survival in hypoxic spheres by downregulating an apoptotic pathway. Further studies are needed to elucidate the prosurvival role of miR-210 on stemness and the effects of directly targeting CASP8AP2 and other proapoptotic proteins in hypoxic EWS spheres. Our data suggest that sphere formation is negatively regulated by CASP8AP2 activity. This is supported in our CASP8AP2 knockdown spheres demonstrating significant increases in sphere formation in the normoxic group and slight increases in the hypoxic group. Furthermore, knockdown of CASP8AP2 significantly decreased apoptosis in normoxic spheres. In our hypoxic spheres, CASP8AP2 correlated with a significant decrease in apoptosis, suggesting that hypoxia induces prosurvival mechanisms that are crucial for sphere formation. Future work will investigate the role of CASP8AP2 and other proapoptotic members involved in regulating survival in hypoxic spheres. Supportive of our findings, previous EWS studies showed that resistance to apoptosis in hypoxic cells was dependent on HIF-1α [39, 40]. Together, these studies indicate that CASP8AP2 silencing by miR-210 prevents apoptosis, promoting sphere formation in hypoxic EWS cells and in normoxic cells pretreated with Hypoxic^EXO^.

This study describes a mechanism whereby EWS cells under hypoxic conditions release exosomes that enhance stemness in EWS cells. We identified a hypoxia regulated miRNA significantly expressed in hypoxic cells and Hypoxic^EXO^ and characterized a potential target that facilitates an apoptotic pathway critical to sphere formation. Ongoing studies are investigating the role of HIF-1α on regulating EWS stemness and together, our future aim is to investigate how HIF-1α selectively modulates the packaging of miRNAs into Hypoxic^EXO^, and validate additional miRNAs that promote aggressive hypoxic phenotypes.

## MATERIALS AND METHODS

### Cell culture and hypoxia exposure

EWS cell lines A673 and SK-ES-1 were obtained from the American Type Culture Collection (ATCC, Manassas, VA, USA). All cells were cultured in RPMI-1640 medium supplemented with 10% fetal bovine serum and 1% penicillin/streptomycin (Invitrogen, CA, USA) as adherent monolayers in a humidity-controlled incubator at 5% CO_2_ and 21% O_2_ at 37°C. Hypoxia experiments for adherent cell growth and sphere formation were performed with a Thermo Scientific™ Heracell™ VIOS CO_2_ trigas incubator (Thermo Fisher Scientific, Waltham, MA, USA) at 5% CO_2_/95% N_2_ and oxygen levels maintained at 1% O_2_ at 37°C. For exosome isolation, A673 and SK-ES-1 cells were cultured in RPMI-1640 media containing 10% exosome-free FBS (System Biosciences, Palo Alto, CA, USA), 1% glutamine and 5% penicillin/streptomycin (Gibco, Carlsbad, CA, USA). In all studies, cell lines were maintained in culture for no more than 10 passages.

### siRNA and miRNA transfection

Pre-designed HIF-1α, CASP8AP2 and Non-Targeting Control (NTC) ON-TARGET *plus* siRNAs in SMARTpool format were obatained from Dharmacon (Horizon Discovery, Waterbeach, UK). miR-210-3p mimics, miR-210-3p inhibitor and negative control (miR-scramble) mimics were purchased from Ambion (Thermo Fisher Scientific, Waltham, MA, USA). All transient transfections were performed with Lipofectamine RNAiMAX (Invitrogen, Waltham, MA, USA) according to the manufacturer’s instructions Following transfections, cells were used in downstream exosome isolations, sarcosphere assays and/or immunoblotting.

### Exosome isolation

Exosomes were prepared from the cell culture supernatant of A673 and SK-ES-1 EWS cells using ultracentrifugation and filtration protocols as described previously [41]. Briefly, EWS cells cultured to 70% confluency were washed with PBS and cultured in RPMI 1640 media containing 10% exosome-free FBS (System Biosciences, Palo Alto, CA, USA) under normoxia (21% O_2_) and hypoxia (1% O_2_) for 48 hrs. The conditioned media was collected and centrifuged for 10 min at 300 × g, 20 min at 2000 × g to remove cell debris, and 30 min at 10,000 × g to remove microvessicles larger than 200 nm. Subsequently, raw exosomes were collected by ultracentrifugation at 100,000 × g for 90 min in a SW 28 rotor (Beckman Coulter, Chaska, MN, USA). Exosome pellets were washed with PBS and passed through a 0.2 μm SFCA filter (Thermo Fisher Scientific, Waltham, MA, USA) to remove large contaminating lipid and vesicular aggregates. This preparation was ultracentrifuged at 100,000 × g for 90 min in a 70.1 Ti rotor (Beckman Coulter, Chaska, MN, USA). Exosomes derived from cells transfected with HIF-1α siRNAs and NTCs were isolated using the Exoquick^™^ reagent (System Biosciences, Palo Alto, CA, USA) with modifications to the vendor instructions. Briefly, conditioned media was centrifuged for 20 min at 2000 × g and 30 min at 10,000 × g. Next, Exoquick^™^ was added to the conditioned media and the remaining exosome precipitation steps were followed according to the manufacturer’s instructions. The exosome pellet was resuspended in 200 μl PBS and used immediately for co-culture assays or stored at –80°C.

### Nanoparticle tracking analysis (NTA)

Exosomes were analyzed using a NanoSight NS300 (NanoSight Ltd, Navato, CA, USA). A red laser beam was used to visualize particles and assess their movement under Brownian motion. Illuminated particles were recorded and analyzed using the NTA software (NTA 3.1 Build 3.1.54) which applies the two-dimensional Stokes-Einstein equation to calculate size distribution and concentration of nano-sized particles.

### Electron microscopy

Transmission electron microscopy (TEM) imaging of exosomes was performed as previously described [42], with some modifications. Briefly, exosomes were fixed for 1 hr in a mixture of 2% paraformaldehyde, 2% glutaraldehyde in 0.1 M Sorensen’s phosphate buffer. Following fixation, the exosome sample was layered on a formvar/silicon monoxide–coated 200 mesh grid and air-dried for 2 min. Grids were then stained with Nanovan negative stain (Nanoprobes, Yaphank, NY, USA) and air-dried for an additional 2 min. Samples were observed at 80Kv with a Tecnai G2 Transmission Electron Microscope (FEI Company, Hillsboro, OR, USA).

### Sarcosphere assay

A673 and SK-ES-1 parental cells and cells transfected with CASP8AP2 siRNAs, NTCs, miR-210 mimics, miR-210 inhibitors and negative control mimics were cultured in serum-free media in non-adherent conditions. To prevent cell aggregations resulting in false positives, cells were seeded at low densities (20 cells/μL) in 6-well or 24-well Corning ultra-low attachment plates in sphere media composed of DMEM/F12 supplemented with 20 ng/mL EGF, 40 ng/mL bFGF, 2 μg/mL Heparin, 0.1 mM β-mercaptoethanol, 1% B27, 1% N2 and 1% penicillin/streptomycin. Sphere formation was assessed, microscopically, in cells grown under normoxia (21% O_2_) and hypoxia (1% O_2_) after 5 days. To assess sphere formation in EWS cells pre-treated with exosomes, EWS cells (1000 cells/well) were cultured at a low density (1 cell/μL) in 24-well low attachment plates with serum-free sphere media (as previously mentioned). Exosomes (20 μg/mL) derived from EWS cells cultured under normoxic and hypoxic conditions were added to A673 and SK-ES-1 cells, respectively. Sarcospheres were counted using a microscope after 5 days. All experiments were performed in triplicate.

For siRNA experiments, exosomes derived from EWS cells transfected with siHIF-1α or NTC were cultured with parental EWS cells and assessed for sphere formation after 5 days.

For miRNA experiments, EWS cells transfected with miR-210 inhibitors were co-cultured with hypoxic exosomes derived from their parental cell line as previously mentioned. Sphere formation and immunoblotting was assessed after 5 days.

### Western blot

EWS cells (2 × 10^4^ cells/well) were seeded in a 6-well ultra-low adherent plate (Corning) and pretreated with exosomes (0 or 20 μg/mL) in a sphere assay. Following 5 days, cells were harvested and lysed for use in western blot. Total protein was isolated from cells and exosomes using RIPA lysis buffer (Thermo Fisher Scientific, Waltham, MA, USA). Protein from cells was quantified using the BCA method (Pierce, Waltham, MA, USA) and the amount of protein in exosomes was estimated by measuring protein content with the Bradford assay (Bio-Rad, Hercules, CA, USA). SDS-PAGE was conducted under reducing or non-reducing conditions followed by immunoblot as previously described [43]. Antibodies used in this experiment were TSG101, CD63, Calnexin (1:500, all Santa Cruz Biotechnology, Dallas, TX, USA), HIF-1α (1:1000, BD Biosciences, San Jose, CA, USA), CASP8AP2/FLASH (1:1000, Abcam, Cambrige, UK) and α-Tubulin (1:4000, Sigma Aldrich, St. Louis, MO, USA). Blots were incubated with appropriate horseradish peroxidase-tagged secondary antibodies and visualized using the _MY_ECL Imager (Thermo Fisher Scientific, Waltham, MA, USA).

### RNA isolation, RNA-seq and analysis

EWS cell and exosome total RNA was isolated using miRCURY™ RNA Isolation Kit according to the manufacturer’s protocol. RNA concentrations and quality were determined by ND-1000 (Nanodrop Technologies, Wimington, DE, USA) and Fragment Analyzer automated CE systems (Agilent, Santa Clara, CA, USA). Eletropherograms were generated using the Fragment Analyzer. Library preparation miRNA-seq used QIAseq miRNA Library Kit (Qiagen, Hilden, Germany), respectively. Sequencing was performed using the Illumina HiSeq3000 system (Illumina, San Diego, CA, USA). The miRNA count was calculated using the QIAseqmiRNA platform. The cutoff of differential miRNA was a fold-change threshold of 1.5 and maximum *p-value* of 0.05.

### Real-time quantitative PCR

For miRNA quantification, miRNA was reverse transcribed and amplified using a Taqman MicroRNA assay kit (Applied Biosystems, Waltham, MA, USA). The Taqman probe based primers included has-miR-10b, has-miR-210 and endogenous controls RNU6B and has-miR-16 (Applied Biosystems, Waltham, MA, USA). miRNA RT-qPCR amplification was performed using the QuantSudio 3 instrument (Applied Biosystems, Waltham, MA, USA). Relative quantification was calculated using a comparartive Ct method.

### Apoptosis assay

To detect apoptosis in EWS spheres transfected with NTC or siCASP8AP2, spheres were dissociated and fluorescently stained using an Annexin-V-FITC/PI flow cytometry assay kit (BD Biosciences, San Jose, CA) according to the manufacturer’s instructions. Quantification of apoptotic cells were performed using the FACSCalibur system (BD Biosciences, San Jose, CA).

### Statistical analysis

Statistical analysis between groups were calculated using Student *t* test or one-way ANOVA with Tukey’s multiple comparisons test. A value of *p* ≤ 0.05 was considered statistically significant. All data is presented as mean ± standard error of the mean (SEM). ΔΔCq method was used to calculate relative gene expression from qRT-PCR data. All experiments were replicated a minimum of three times unless otherwise indicated.
